# The Autonomous Pipeline Navigation of a Cockroach Bio-Robot with Enhanced Walking Stimuli

**DOI:** 10.34133/cbsystems.0067

**Published:** 2023-11-08

**Authors:** Songsong Ma, Yuansheng Chen, Songlin Yang, Shen Liu, Lingqi Tang, Bing Li, Yao Li

**Affiliations:** ^1^ State Key Laboratory of Robotics and System, Harbin Institute of Technology, Harbin, China.; ^2^ Guangdong Key Laboratory of Intelligent Morphing Mechanisms and Adaptive Robotics, Harbin Institute of Technology, Shenzhen, China.; ^3^ School of Mechanical Engineering and Automation, Harbin Institute of Technology, Shenzhen, China.; ^4^ The Biorobotics Institute, Scuola Superiore Sant'Anna, Pisa, Italy.

## Abstract

Tens of crawling bio-robots with cockroaches as the mobile platform have been developed with various functions. Compared with artificial crawling robots of the same size, they revealed better flexibility, larger payload, and stronger endurance. These features made bio-robots ideal for pipeline inspection scenarios because the advancements in locomotion mechanisms and efficient power systems are still hurdles for current artificial systems. In this study, we controlled the bio-robot to crawl in the confined dark pipeline and achieved autonomous motion control with the help of an onboard sensing system. Specifically, a micro-camera was mounted on the electronic backpack of the cockroach for image collection, and an IMU sensor was used to compute its body orientation. The electronic backpack transmitted images to the host computer for junction recognition and distance estimation. Meanwhile, the insect's habituation to electrical stimulation has long been an uncertain factor in the control of bio-robots. Here, a synergistic stimulation strategy was proposed to markedly reduce the habituation and increase the number of effective turning controls to over 100 times. It is also found that both the increase of payload and the application of stimulations could promote the metabolic rate by monitoring carbon dioxide release. With the integration of synergistic stimulation and autonomous control, we demonstrated the fully autonomous pipeline navigation with our cockroach bio-robot, which realized the cycle number of approximately 10 in a roll. This research provides a novel technology that has the potential for practical applications in the future.

## Introduction

Bio-robotics is an emerging field with ongoing research and development. The bio-robot combines the features of the live creature with artificial elements to control its natural behaviors. The bio-robot reveals higher flexibility and energy efficiency than state-of-the-art artificial miniature robots. Thus, they can leverage the advantages of both biological and artificial systems, with applications ranging from environmental surveillance to search and rescue missions, and potentially even targeted delivery of payloads in the narrow spaces that humans cannot reach. It is a significant area of research that holds promise for various scientific discoveries and practical applications.

The development of artificial centimeter-size robots requires a complicated mechanism design for multiple degrees of freedom of motion and precise manufacturing processes. Moreover, robots with high degrees of freedom need complex controls, such as the hexapod HAMR, which uses different gaits to achieve forward locomotion and zero-radius turns [1]. On the contrary, the bio-robot only needs to implant electrodes and apply electrical stimulation signals at the locations of nerves and muscles related to movement. Then, the small creatures can be transformed into a controllable mobile platform. For example, the turning of the cockroach [2,3], the beetle [4], and the locust [5,6] was induced by applying electrical stimulation signals on the contralateral antenna; the steering flight of the honeybee was initiated by stimulating the nervous system near the compound eyes [7]; the direct muscle contraction was controlled by electrical stimulation on the muscles of the beetle [8–10], the locust [11–13], and the moth [14,15]. Artificially cultured muscle tissue could make smaller bio-actuators. Zhang et al. [16] made a manta ray-inspired biosyncretic swimmer and improved its stability. Morimoto et al. [17] made a ~16-mm muscle-driven robot to pick small objects.

The motion of both bio-robots and micro-robots is mostly manually controlled. For the few robots that achieved feedback control, an external motion capture system with attached markers was widely used, which made them unattainable for navigation in confined environments. For example, the Microsoft Kinect was used for image processing and positioning of bio-robots, which can be used to control robots crawling along preset trajectories [18]; a camera and augmented reality (AR) marker were used to track and control the movements of the cockroaches in a real-time way [19]; a swarm of cockroaches in conjunction with an aerial leader achieved exploration and mapping of the unknown environment [20]. However, with the absence of the external positioning device, it is difficult for the robot to locate the target. Using an onboard microphone array or acoustic sensors attached to a bio-robot was a rare way to achieve a target search. This bio-robot can only move in the direction of the sound source and can not observe the surroundings in the dark [21,22].

The development of bio-robots with cockroaches has gained continuous attention in the past 20 years. The cockroaches have large payload capacity, excellent crawling capability, and electrical signal sensitivity, making them ideal moving platforms for bio-robots. Equipped with sensors such as cameras, microphones, and gas detectors, they can be used for pipeline inspection missions. The existing man-made pipeline robots can usually be divided into 3 categories: wheeled wall-pressed type, caterpillar wall-pressed type, and wheeled wall-pressing screw type [23]. They need to be wired for power and signal transmission. To ensure their versatility, they cannot be further reduced in size. Thus, the use of bio-robots can minimize the size of the robot for smaller pipes. Moreover, the power consumption of bio-robots is extremely low, ranging from 100 to 1000 mW [24].

**Fig. 1.**
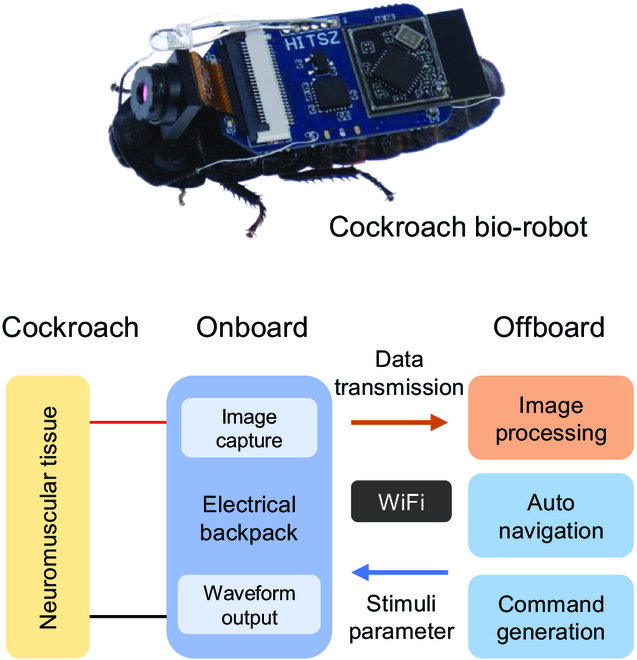
The appearance of the cockroach bio-robot. The bio-robot system consists of the insect platform, the electronic backpack, and the commanding software.

In this research, we designed a cockroach bio-robot that enabled long-acting crawling control and autonomous navigation in the confined dark pipeline, as shown in Fig. 1. Specifically, based on the existing unipolar pulse stimulation on antennae and cerci, a new synergistic electrical stimulation strategy was proposed to prolong the effective electrical stimulation trials. With the help of the onboard camera and inertial measurement unit (IMU), the bio-robot can adjust its orientation and perceive the distance to the junction in a pipeline.

## Materials and Methods

### Electrode implantation and turning speed measurement

Female Madagascar hissing cockroach (*Gromphadorhina portentosa*) (length: 5.5 ± 0.3 cm, mass: 6.1 ± 1.1 g) was selected for the experiments. All the cockroaches were bought from local pet stores and kept in cages in small groups (~5 cockroaches). They were fed with insect feed once a week. The temperature was set to 25 °C, and the moderate setting was 60%. The use of cockroaches was permitted by the Animal Ethical and Welfare Committee (IACUC-2020026).

**Fig. 2.**
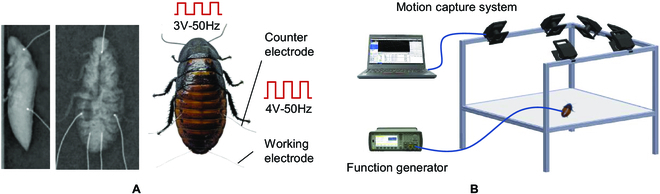
The electrode implantation and turning test. (A) Electrode implantation under x-ray. The working electrodes of the antennae and the cerci were implanted to a depth of 3 and 10 mm, respectively. The corresponding counter electrodes were burned to form a small ball at the tip and then implanted into the surface of the head and the third abdominal segment. (B) A function generator was used to produce control signals, and a 3D motion capture system was used to analyze turning speed.

The antennae and the cerci were both implanted with electrodes to control the cockroach's turning. The cockroach was anesthetized with carbon dioxide gas for 1 min. For antennae electrical stimulation, the working electrodes (76.2 μm uncoated diameter, 139.7 μm coated diameter, A-M Systems) were implanted to a depth of 3 mm after the antennae have been cut to retain a length of 10 mm. The counter electrode was implanted in the head at a depth of 1 mm. For cerci electrical stimulation, the cerci tip was cut, and the working electrode was implanted at a depth of 10 mm. The counter electrode was implanted 1 mm deep into the skin of the third abdominal segment. Specifically, the tip of the counter electrode was burned into a small ball and implanted under the exoskeleton for better fixation and better stimulation. Electrical stimulation of the antennae causes the cockroach to turn in the direction opposite to the electrical stimulus, while electrical stimulation of the cerci can make the cockroach turn in the same direction as the electrical stimulus. The voltages of antennae and cerci were 3 and 4 V, respectively. The frequency and duty cycle of electrical stimulation were 50 Hz and 50%, respectively, as shown in Fig. 2A.

The turning speeds were obtained with the help of the three-dimensional (3D) motion capture system (6 × Vicon V5 cameras; Oxford Metrics Ltd., Oxford, UK) at 500 frames per second (fps), as we can see in Fig. 2B. Two 3-mm-diameter retro-reflective markers were taped to the axis of the body on the back of the cockroach. A function generator was used to produce the desired signals. The single measurement time of the motion capture system was 2.5 s. The first 1 s was the time before stimuli, which was used to calculate the angular velocity reference; the output of the stimulation signal lasted 1.5 s, which was used to obtain the rotational angular velocity of the cockroach during electrical stimulation. Different time intervals between 2 stimulations (15, 60, and 120 s) were set to obtain the required measurement data. The turning speed was calculated in the same way as Erickson's study [25]. Moreover, a synergistic stimuli method was proposed in this study. Electrical stimulation signals were applied to the cockroach by alternating the stimulation sites between the antennae and cerci. Thus, the neuromuscular were not continuously stimulated and had longer rest time to recover. In the synergistic stimuli test, the time interval between 2 adjacent stimulations was set to 10 s. The alternation sequence was set to every 2 stimulations or every 5 stimulations on the same site. Then, the turning velocity was measured and compared with that of the antennae or cerci stimulation.

### Design of the electronic backpack

The electronic backpack has an envelope size of 45 mm × 18 mm × 7 mm and a total mass of 5.5 g, including a 2.5-g battery. The chip is STM32H743VIH6, which has a small package with an integrated JPEG hardware codec, DCMI interface, 2-Mb flash, and a processing core with a frequency of up to 48 MHz. This chip can be ported FreeRTOS and can process information while ensuring a timely response to motion control commands. A 24P 0.5-mm FPC connector on the electronic backpack enables the connection of a miniature camera (OV2640). The electronic backpack can shoot videos at a frame rate of 6 fps, with a single image resolution of 320 × 240. The WiFi module with built-in TPC communication protocol can achieve high-speed wireless communication within 15 m.

### Metabolic measurement of cockroaches

After installing the electronic backpack on the back of the cockroach, the metabolic rate of the cockroach was measured under the control of the electronic backpack. The metabolic was estimated from carbon dioxide production. The Qubit system (Q-Box RP1LP Low Range Respiration Package, Canada) was used to measure carbon dioxide production. The bio-robot was placed in a chamber for crawling. Metabolism in the states of resting, crawling without a backpack, and crawling with a backpack were also measured in the control groups. Non-electrically stimulated crawling was initiated by swaying the chamber back and forth slightly.

### Crawling control in the confined pipeline

**Fig. 3.**
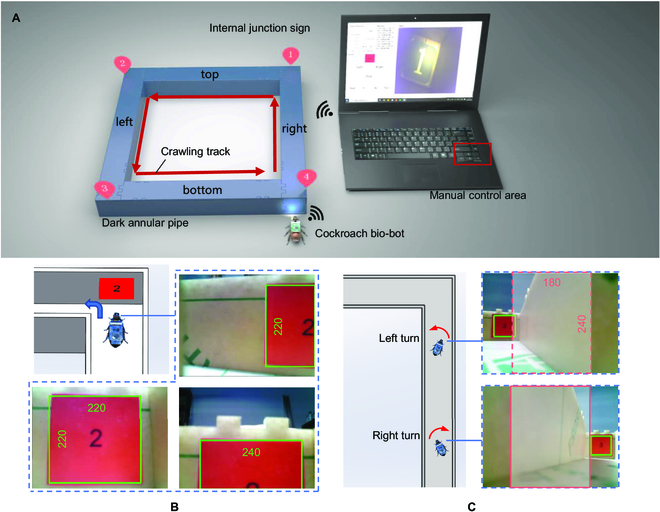
Diagram of a controlled cockroach bio-robot crawling through a dark confined pipeline. (A) A cockroach bio-robot equipped with LED was connected to the host computer via WiFi and transmitted videos from the pipeline to the host computer. The manual or autonomous control signal was sent from the host computer to the bio-robot to control its crawl through the pipeline. (B) Identification of the red square sign in the junction via image processing at the host computer. Left turning control was triggered when the identified outline was equal to or greater than 220 pixels. (C) When the bio-robot was approaching a junction, a rectangle box of 180 × 240 pixels was set on the image for directional calibration. Once the outlines of the junction sign were outside the rectangular box, a 0.2-s electrical stimulus was applied to correct the crawling direction.

A confined square pipeline with a width of 65 mm was designed to verify the control of the bio-robot, as shown in Fig. 3A. This size was wide enough and did not restrict the cockroach's turnaround. Four red square signs (30 mm × 30 mm) were attached to the junctions and recognized on the host computer. A window (320 × 240 resolution) was used to display the video returned from the electronic backpack and the identification of the red square sign. The upper cover of the pipeline can be removed for an open bright environment. For manual control in bright environments, the operator applies the appropriate electrical stimulus by directly observing the position of the bio-robot in the pipeline. For manual control in confined dark environments, the light-emitting diode (LED) on the electronic backpack lighted up the pipeline and the operator controlled the bio-robot via video from the host computer. The average crawling speed of each path segment was calculated with the help of a time recorder. The turning speed was judged from the video recording, using the time between the start of the stimulated deflection and the completion of the quarter turn.

Apart from the manual control, the autonomous navigation of the bio-robot in the pipeline was conducted via image processing. The images were converted into a hue saturation value (HSV) color model, and the red HSV range was set. We defined the red HSV range, created a mask, and extracted the red area. Then, we performed morphological operations, including filling holes, smoothing edges, finding the outline in the image, traversing the outline, and filtering for square outlines that fit within the color threshold range.

The recognized outline of the red square signs can be used to determine the distance of the bio-robot from the junction and the orientation of the bio-robot body. According to the experimental test, when the width or the height of the sign was greater than 220 pixels, the bio-robot reached the junction, and a 1-s left turn signal was applied on the bio-robot, as shown in Fig. 3B. When the IMU detects a turning angle greater than 70 degrees, the turning signal stops; otherwise, an additional 1-s turning signal was applied. The width of the square pipeline was greater than the length of the bio-robot. Inevitably, the bio-robot would deflect or even turn around as it crawls forward. Thus, the walking direction was maintained by the camera and the IMU. Specifically, we introduced the directional calibration when the bio-robot was approaching a junction. We defined a 200 × 240 pixel area in the center of the image. When the recognized outlines of the red square sign were outside the defined area, the opposite 0.2-s reverse turning control was applied so that the outlines fell within the defined area, as shown in Fig. 3C. The proposed synergistic electrical stimulation method was fully utilized, with the antennae and cerci exchanging electrical stimulation positions every 5 trials. Thus, the autonomous navigation of the bio-robot in the pipeline was performed.

## Results and Discussion

### Long-acting turning control by synergistic stimuli

**Fig. 4.**
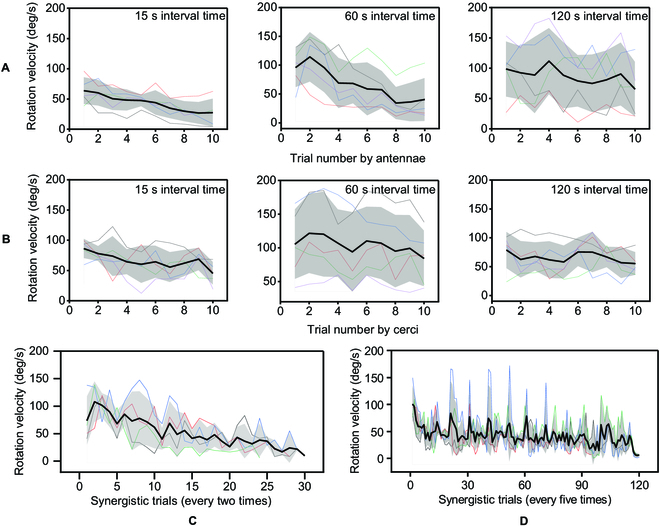
Turning control by synergistic stimuli. (A) Attenuation of turning speed based on antennae electrical stimulation with 15-, 60-, and 120-s intervals. The corresponding attenuation percentage after 10 electrical stimuli were 57.2%, 57.9%, and 33.6%, respectively. (B) Attenuation of turning speed based on cerci electrical stimulation with 15-, 60-, and 120-s intervals. The corresponding attenuation percentage after 10 electrical stimuli were 47.5%, 20%, and 30%, respectively. (C) Alternating electrical stimulation sites between antennae and cerci every 2 trials. The turning speed became less than 30 deg/s after 29 trials. (D) Alternating electrical stimulation sites between antennae and cerci every 5 trials. This configuration can effectively turn the cockroach more than 100 times. *N* = 5 cockroaches and *n* = 5 trials.

Continuous short-interval electrical stimulation could induce fast habituation of electrical stimulation on antennae. Under continuous antennae electrical stimulation with 15- and 60-s intervals for 10 trials, the turning speed was significantly reduced and the corresponding values were 57.2% and 57.9%. However, with the 120-s interval, the reduction in turning speed was much less significant, only 33.6%, as shown in Fig. 4A.

During cerci control, using our electrode implantation and electrical stimulation allows the cockroach to gain turning control ipsilateral to the electrical stimulation side. The electrode implantation positions were the same as Tran-Ngoc's [26] research, while their depth of counter electrode implantation was 5 mm. Different implantation depths allow the current to flow through different neuromuscular tissues, obtaining opposite directions of turning. When the time interval was set to 15 s, the turning speed was reduced by 47.5%. While for 60- and 120-s intervals, the turning speed only reduced by 20% and 30%, respectively, as shown in Fig. 4B.

For most studies, turning control in cockroaches was achieved through antennae, which was convenient and stable [3,21,25,27,28]. But as the demands on cockroach-based mobile platforms grew, the use of cerci control emerged, which retained the perception functions of the antennae. For example, in Kakei et al.'s research [2] and Tran-Ngoc's research [26], this method preserved the detection function of the antennae. However, both control methods of the cockroaches became habituated after multiple electrical stimulations, and a long rest was required to regain the sensitivity of neuromuscular tissues to electrical stimulation.

As for the proposed synergistic stimuli, alternating the position of electrical stimulation could help prolong the effective stimulation trials. The alternation of every 2 trials could effectively turn the cockroach 29 times. The turning speed also showed the same gradual decay similar to that controlled by the antenna or the cerci only. In the 30th control, the turning speed was decreased to only 9.7 deg/s, as shown in Fig. 4C. After increasing the alternation number to 5 trials per site, it was possible to maintain a turning speed of above 45 deg/s with fluctuations, and the number of effective controls exceeded 100, as shown in Fig. 4D.

Increasing the time interval between 2 electrical stimulations at the same position is an effective way to reduce this control attenuation. Increasing the interval of antennae/cerci electrical stimuli to 120 s/60 s can make the turning speed decay by only about 30% in the first 10 trials. Both the antennae and the cerci have sufficient time to recover their sensitivity to electrical signals. The essence of the synergistic electrical stimulation is also to give the antennae and the cerci more rest. Switching positions every 2 electrical stimulation gives the antennae or cerci a 23-s rest, and increasing the frequency to 5, the antennae or cerci will have a 57.5-s rest. The neuromuscular tissues can regain their sensitivity to electrical signals by relying on the organisms' regulation. Unlike the control of muscles by nerve signals, functional neuromuscular stimulation is a charging process for the cockroach, which indeed affected the motion control of the artificial electrical stimulation signal. Neuromuscular stimulation with bipolar pulses enables zero charges. The first negative pulse causes depolarization of the nerve fibers, and the second positive pulse acts as a charge balance [29,30]. Specifically, a bipolar pulse with an exponential decay positive waveform, which is called Lilly waveform, is widely used in no-charge stimulation [31]. However, the size of the electronic backpack does not allow for the addition of more peripheral circuits to output bipolar pulses. It is much easier for a miniature electronic backpack to emit unipolar pulses. Therefore, for miniature electronic backpacks, synergistic electrical stimulation is an effective method for prolonged control of cockroach bio-robots.

### Crawling control in the confined pipeline

**Fig. 5.**
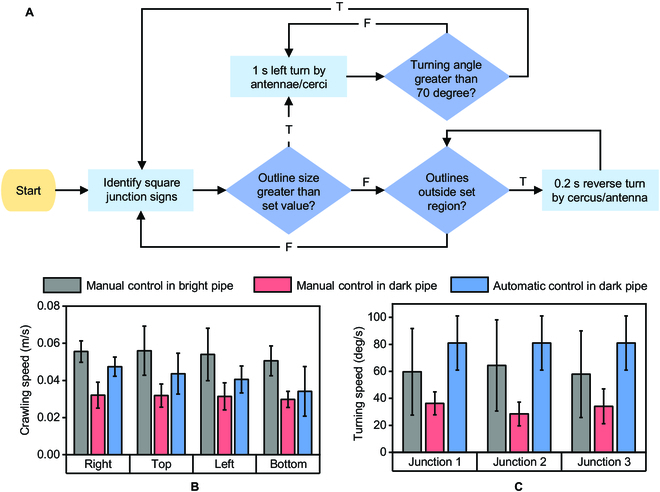
Comparison of manual control and autonomous control in the confined pipeline. (A) Flow chart for autonomous navigation control of a cockroach bio-robot. The host computer identified the junction sign for each frame returned from the bio-robot. A judgment condition was used to control the bio-robot's turning at a junction, and another judgment condition was used to correct the deflection of the crawling direction before approaching a junction. (B) Comparison of the bio-robot's crawling speed through 4 segments by manual control in the open bright pipeline, manual control in the dark confined pipeline, and autonomous control in the dark confined pipeline (*N* = 5 bio-robots, *n* = 5 trials). The corresponding values were about 0.055, 0.03, and 0.04 m/s. (C) Comparison of the bio-robot’s turning speed at the junctions in 3 different control environments (*N* = 5 bio-robots, *n* = 5 trials). The corresponding values were about 60, 33, and 80 deg/s.

The cockroach bio-robot can be controlled to crawl through dark confined pipeline using both manual and autonomous methods. The forward crawling and turning speeds were relatively constant in the 3 control methods. The bio-robot can reach its fastest crawling speed (0.055 m/s) in the open bright environment by manual control. Using the control flow diagram in Fig. 5A and appropriate electrical stimulation signals, the autonomous navigation in the dark confined pipeline reached the speed of 0.04 m/s. The slowest manually controlled crawl under dark confined pipes was only 0.03 m/s. By the crawling speed, the turning speed in the bright (60 deg/s) was greater than that in the dark (33 deg/s) under manual control. However, the turning speed obtained by the autonomous navigation in the dark was faster than both manual controls, which was 80 deg/s.

For manual control in the dark confined pipeline, the LED can only illuminate a limited area of the pipeline, and the frame rate of the video was only 6 fps, which causes the operator to take more time to determine the position of the bio-robot, therefore making control slower. For autonomous control in the dark confined pipeline, the forward crawling was controlled by a stimuli signal every 5 s. The electrical stimulation signal was not applied in time when the crawling speed of the bio-robot slowed down. While for manual control in the open pipeline, by directly observing the position of the bio-robot in the pipeline, the operator can control a faster crawling speed by applying a forward in time, which contributed to the fast crawling speed.

The turning control in 3 cases had different effects. For manual control, both in the open bright pipeline or confined dark pipeline, control signals were applied based on visual feedback from the operator, and it was difficult for a human to apply a fixed time signal to a bio-robot. The operator applied too long or too short a duration of electrical stimuli, and can not accurately determine the distance of the bio-robot to the junction, making the turning angle too large or too small and slowing down the turning. For the autonomous turning control, 1- or 2-s continuous stimuli were applied under the set judgment condition (the identified outlines and the IMU data). The automatic method has advantages for controlling the turning of cockroach bio-robot.

**Fig. 6.**
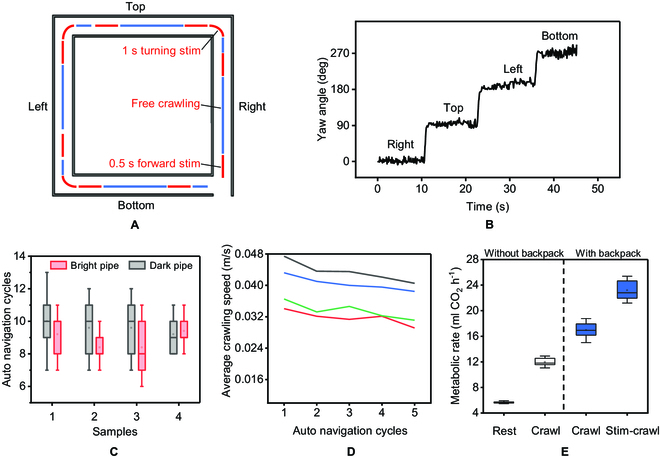
Autonomous navigation in the pipeline environments. (A) Representative diagram of electrical stimuli application for autonomous control. (B) Change of IMU data when the cockroach bio-robot crawled in the corresponding path. (C) The navigation performance in bright and dark environments was compared. The brightness did not affect autonomous control in the pipeline. The average navigation distance was about 10 cycles (*N* = 4 bio-robots and *n* = 20 trials). (D) The autonomously controlled crawling speed was gradually reduced concerning crawling cycles. (E) The metabolic rate of cockroaches increased in the 4 states, namely, resting and crawling without a backpack, crawling, and stimuli crawling with a backpack (*N* = 5 bio-robots and *n* = 20 trials).

Representative signals for bio-robot's autonomous control to complete a cycle of crawling are shown in Fig. 6A. A 0.5-s forward stimulation was applied to force the bio-robot to crawl, and after 5-s free crawling, another 0.5-s forward stimulation was applied to the cerci. When the bio-robot arrived at the junction, 1-s turning stimulation can turn the bio-robot to the next path segment. Typically, a 1-s electrical stimulation produced an approximately 90-degree turn, reducing the need for a second steering adjustment. The turning at the junction was sensed by the IMU on the backpack of the cockroach bio-robot as in Fig. 6B. The cockroach's body swung slightly with the movement of its legs during crawling. This straight-line crawl can also be seen in Movie S1. Seldomly, the cockroach produced significant directional deflections during their straight-line crawls. Then, the IMU data were used for course correction. Autonomous navigation of the bio-robot was tested in both bright and dark pipes. Four samples can complete about 10 cycles of autonomous navigation in both environments (*N* = 4 bio-robots, *n* = 20 trials), as shown in Fig. 6C. We also calculated the average crawling speed in the first 5 cycles. They all experienced a slight attenuation, as shown in Fig. 6D. However, as a comparison, the bio-robot without our control strategy can hardly finish a single cycle of pipeline crawling (0 of 10 trials completed).

Metabolic rate is an expression of energy expenditure in cockroaches and can be measured by the production of carbon dioxide. At resting and crawling without a backpack, carbon dioxide is produced at a rate of 5.7 and 12 ml/h, respectively. After installing an electronic backpack with a mass approximately equal to its weight, the rate of carbon dioxide production has increased significantly. In free crawling and stimuli crawling, the values were 17 and 23.2 ml/h, respectively. The metabolic rate of stimuli crawling with a backpack was about 2 times of the free crawling without a backpack, as shown in Fig. 6E.

The walls of the pipeline can have a limiting effect on the crawling of the bio-robots. The autonomously controlled directional correction in forward crawl is only occasionally required in practice. This method can only be used in bright environment. In the dark pipeline, the LED light is not sufficient to identify junction signs at long distances. In the absence of applied turning control, the bio-robot crawled in a near straight line [28]. Although large angular deflections or even complete turning rarely occurs, it is still a critical technology in practice and new image processing techniques need to be developed to provide control of deflection correction.

Energy expenditure is one of the reasons for slowing down the controlled crawl. The weight bearing increased the metabolic rate of the cockroach. The total weight of the backpack was 5.5 g, which was almost the same weight as the selected cockroach (6.1 g). The cockroach needs more energy to load an electronic backpack close to their weight. However, for the same electronic backpack fitted, the metabolic rate was significantly higher for locomotion under electrical stimulation than under non-electrical stimulation. The electrical stimulation signal causes the cockroach to accelerate instantaneously, and the rapid muscle contraction needs to provide more energy. Also, the electrical signal itself can accelerate the metabolism of cockroaches. Previous studies have proved that electrical stimulation can enhance the force generation and metabolic flux of the engineered human muscle [32]. Also, functional electrical stimulation can increase oxidative enzymes in both humans and animals [33]. The other reason for slowing down the controlled crawl was the overuse of the cerci. The cerci were used not only in the synergistic turning control but also in the forward control, leaving insufficient time for the cercus to recover. A lighter electronic backpack and more forward control methods need to be further developed in future research.

## Conclusion

In this research, we demonstrated a cockroach bio-robot capable of navigating autonomously in the pipeline. A synergistic electrical stimulation method of the cockroach's antennae and cerci was proposed, which can extend the effective control trials to over 100 times. Meanwhile, a LED installed on the electrical backpack can provide a light source in the dark pipeline. In the dark confined pipeline, the bio-robot's crawling speed was ~0.04 m/s, and the turning speed was ~80 deg/s in the autonomous navigation. Moreover, the brightness did not affect autonomous control in the pipeline apparently. The average navigation distance in the dark pipeline and bright pipeline was about 10 cycles, which went through ~40 internal junctions. However, it is found that the application of electrical stimulation could increase the metabolic rate of the cockroach. The number of stimulation should be reduced to elongate the crawl distance. With the findings of this work, we believe the cockroach bio-bots are one step closer to practical application.

## Data Availability

All data are available from the corresponding author upon reasonable request.
